# Supplementary material for: ERG-driven prostate cancer initiation is cell-context dependent and requires KMT2A and DOT1L
Source: Nat Genet. 2025 Aug 26;57(9):2177–91. doi: 10.1038/s41588-025-02289-w (PMC12425824; doi:10.1038/s41588-025-02289-w)
Supplement: Supplementary file 1 — Supplementary Notes 1–8, Fig. 1 and References. [file 41588_2025_2289_MOESM1_ESM.pdf]

nature genetics

Supplementary information https://doi.org/10.1038/s41588-025-02289-w

ERG-driven prostate cancer initiation is cell-context dependent and requires KMT2A and DOT1L

In the format provided by the authors and unedited

Table of Contents

This file includes:

Supplementary Notes 1-8

Supplementary Fig. 1

Supplementary References

Additional Supplementary Files not included here:

Supplementary Tables 1-6

Supplementary Note

Supplementary Note 1. Skin phenotypes in EP mice crossed to Basal Cre drivers.

Having implicated basal cells as cell of origin in this transplantation assay, we turned to conventional lineage tracing approaches to validate our findings in an autochthonous model using a tamoxifen-inducible *K5-CreER*^{T2} (basal) mouse strain, a well-established tool to address lineage questions in various epithelial tissues[1-4]. After confirming the lineage fidelity of this strain in prostate by crossing to a YFP reporter strain (**Extended Data Fig. 1D**), we generated the relevant *EP* compound mice (*EP*; *K5-CreER*^{T2}), administered tamoxifen at age 7-9 weeks, then followed mice for disease onset. However, we were unable to score the prostate phenotype because all mice had to be sacrificed within 2-3 weeks of Cre induction due to a fulminant skin disease with extensive desquamation. We observed an essentially identical phenotype using an independent basal-specific Cre driver strain (*K14-CreER*) (**Extended Data Fig. 1E-J**) but not in *Pten*$^{flox/flox}$ mice, suggesting that it is a consequence of aberrant expression of ERG in K5/14-expressing cells in skin[5].

Supplementary Note 2. Recombination efficiency of Cre drivers.

To determine whether differences between Cre drivers may confound interpretation of the phenotypes, we compared recombination efficiency using YFP and ERG as readouts. Although less efficient than Nkx3-1-CreER^{T2}, K8-CreER^{T2} was active (based on YFP expression) across distal regions of all three lobes, similar to Nkx3-1-CreER^{T2}, and additionally in proximal regions where *Nkx3-1* is not expressed[6] (**Extended Data Fig. 4A-B**). Furthermore, the level of ERG expression was comparable across the two Cre drivers within in the recombined population, as expected by use of the universal Rosa26 promoter (**Extended Data Fig. 4C**). In addition, luminal cells in the prostates of *EP;K8-CreER*^{T2} mice had reduced *Nkx3-1* expression within a month of tamoxifen induction (**Extended Data Fig. 4D)**, likely reflecting transition to a stem-like (L2) luminal state as a consequence of Pten loss (discussed further below, **Fig. 4**). Although we cannot exclude Cre recombination efficiency as a variable, the collective evidence suggests that the lack of invasive phenotype seen in *K8-CreER*^{T2}*;EP* versus *Nkx3-1-CreER*^{T2}*;EP* mice is explained by a preferred basal cell of origin.

Supplementary Note 3. IM cells in the *EPC* and *PC* models.

We next extended our analysis of IM cells to our original *EPC* model, where ERG is expressed in both basal and luminal cells in a direct comparison with *WT* mice (*Rosa26-YFP$^{LSL/LSL}$;Pb-Cre4*, i.e. *YC*), and to mice with prostate specific *Pten* deletion alone (*Pten$^{flox/flox}$;Rosa26-YFP$^{LSL/LSL}$;Pb-Cre4*, i.e. *PYC*). As expected, prostates from *WT* mice had two major epithelial populations, single-positive luminal cells (K8+/K18+) and single-positive basal cells (K5+/p63+) with rare (~1%) double-positive cells. In contrast, *PYC* and *EPC* mice had expanded IM populations, with reduced expression of basal master regulator p63 relative to *WT* basal cells (**Fig. 2I, Extended Data Fig. 5D-E**). Consistent with the other models, ERG+ IM cells in EPC mice are highly proliferative (30% Ki67+), morphologically luminal and co-express K5, K8/18 and androgen receptor (Ar) (**Fig. 2J-K, Extended Data Fig. 5F-H**). We also found a substantial fraction of K5-negative, K8+ luminal cells with regions of invasive adenocarcinoma (**Fig. 2I, Extended Data Fig. 5F-G**), consistent with the phenotype of ERG+ human tumors.

To distinguish between the effects of ERG versus *Pten* loss on expansion of IM cells, we performed similar tamoxifen-induced lineage tracing experiments following basal- or luminal-specific Pten deletion only (no ERG). Highly proliferative IM cells were detected 3-6 months following Tam administration to *PY;K5-CreER^{T2}* mice but not *PY;K8-CreER^{T2}* mice, indicating the IM state occurs only after *Pten* deletion in basal cells and is not ERG-dependent (**Extended Data Fig. 5I-K)**. As expected[7,8], no cell fate changes were observed in wild-type basal cells (*Y;K5-CreER^{T2}*). In contrast to the basal-dependence of the IM state, the histologic phenotype of intraductal hyperplasia typically associated with *Pten* loss was equally evident following basal or luminal-specific deletion (**Extended Data Fig. 5L**). We conclude that *Pten* loss in basal cells is sufficient to generate the highly proliferative IM state, whereas ERG is required for invasive adenocarcinoma.

Supplementary Note 4. ERG-driven basal-IM-luminal lineage transition in organoids.

To gain greater clarity into these lineage transitions, we moved from EPC mice to a mouse organoid system that allows direct tracing of lineage fate. After establishing primary prostate organoids from $ERG^{LSL/LSL}$; $Pten^{flox/flox}$ mice, we infected the organoids with Cre virus to delete Pten and activate ERG expression (**Extended Data Fig. 6D-E**), then repeated the EdU pulse chase experiment but now *in vitro*. After the 2 hour pulse, the EdU label was primarily in basal cells (>80%) but shifted to DP and SP^{lum} cells during the chase (35% and 27% respectively) and was ERG-dependent (**Fig. 3D-F, Extended Data Fig. 6F**). The above experiment demonstrates the pro-luminal consequences of ERG induction in a mixed culture of basal and luminal cells.

To ask if ERG can drive a luminal fate transition from purified basal cells, we generated a dual reporter system in *EP* organoids by knocking fluorescent marker genes into the endogenous *Krt5* and *Krt8* loci (**Extended Data Fig. 6G-L**), thereby providing a platform to isolate live basal or luminal cells for subsequent lineage commitment studies. After confirming the expected localization of the respective K5-targeted (basal) and K8-targeted (luminal) fluorescent signals (**Fig. 3G**), we tracked the lineage fate of sorted basal and luminal cells in organoid culture. ERG-positive basal cells showed a ~5-fold increase in luminal fate transition compared to ERG-negative cells (**Fig. 3H**, left panel**)**, confirming that ERG accelerates luminal differentiation from basal cells. Transcriptomic analysis confirmed ERG-dependent activation of luminal gene signatures in basal cells (**Fig. 3I**, **Supplementary Table 1**). In contrast to the increase in luminal cell output from sorted basal cells, ERG was not sufficient to sustain luminal fate in sorted luminal cells (**Fig. 3H**, right panel). This likely reflects the fact that the organoid culture conditions used here are optimized to support self-renewal (through inclusion of WNT ligands such as R-spondin) and subsequent lineage commitment rather than sustained growth or persistence of fully differentiated luminal cells. Indeed, previous single cell analyses of murine prostate organoid cultures have shown enrichment of stem-like (L2) luminal cells and near absence of secretory (L1) luminal cells[9]. Taken together, the EdU and lineage tracing studies provide evidence that ERG-driven cancers initiate in basal cells, which expand initially as highly proliferative IM cells then transition to luminal cells.

Supplementary Note 5. IM cell state in ERG+ human prostate cancer.

To determine whether the expanded IM population seen in GEMMs is seen in human prostate cancer, we compared gene sets identified through single cell analysis of our EPC mice

with single cell data from ERG+ human prostate cancer. Elevated MYC, NF-κB, RNA processing and translation transcriptional programs were enriched in both mouse and human (**Fig. 4G-H**), suggesting shared mechanisms across the two species. Moreover, several genes that define IM cells in mouse (Pmepa1, Mif, ApoE, S100a11, Sfn, Anxa1) are expressed at elevated levels in human prostate cancer (versus normal prostate) or in ERG+ (versus ERG-) human prostate cancers, even in bulk RNA-seq data where the relative abundance of IM cells is likely low (**Extended Data Fig. 8A**).

Supplementary Note 6. TF motif enrichment in PC-IM and EPC-IM clusters.

TF motifs enriched in both PC-IM and EPC-IM clusters (category one) include NF-κB (NFKB2, REL) and AP-1 (FOSL1 and others), consistent with prior work implicating both pathways in prostate and other cancers[10-16]. Because PC-IM cells arise earlier than EPC-IM cells in pseudotime, we postulate that PTEN loss is responsible for NF-κB and AP-1 family TF activation in IM cells in both *PC* and *EPC* mice.

For the second category of EPC-IM specific enrichment, we identified STAT and NFAT family TFs, all with a high stringency threshold (adjusted p-value $< 1x\ 10^{-20}$). Based on the correlation of motif accessibility and inferred gene expression, STAT3 and NFATC1 are implicated as the top candidates for each family and, consistently, their motif accessibilities were increasingly enriched across pseudotime only in EPC-IM cells, whereas NFκB2 motif enrichment occurs in PC- and EPC-IM cells (**Fig. 6E**). Because STAT and NFAT TFs play critical roles in driving progenitor and inflammatory programs in other tissue contexts including cancer[17-20], it is plausible that STAT3 and NFATC1 may also contribute to the ERG-induced stem and inflammatory programs identified earlier (**Fig. 4E**). It is worth noting that STAT3 and NFATC1 are expressed in IM cells regardless of ERG status; however, the accessibility of their binding sites is significantly higher when ERG is expressed, implicating cooperativity for enabling accessibility (**Fig. 6F, Extended Data Fig. 9K**).

Supplementary Note 7. Cell context dependencies of ERG.

Our finding of a restrictive cell context for initiating ERG-driven prostate cancer in the mouse raises intriguing questions about the human context, where translocations into the *TMPRSS2* locus can presumably occur in relatively rare Basal^{Lum} cells as well as in highly

abundant canonical luminal cells. It is widely accepted that ERG status alone is not prognostic[21-26]; however, our mouse data suggest a context where ERG could be prognostic depending on the cell type in which the initial translocation arises. Specifically, translocations arising in $Basal^{Lum}$ cells would be expected to give rise to high grade adenocarcinomas, whereas those arising in canonical luminal cells might be unproductive or generate lower grade carcinomas[27-34]. We report some evidence in support of this hypothesis by inferring cell of origin through analysis of bulk RNA-seq data from existing clinical cohorts (using IM versus luminal signatures) (**Fig. 5C**) although these results are limited by the retrospective nature of this work. Future studies using prospective cohorts with single cell data are needed to provide further insights.

More broadly, the fact that ERG requires such precise cellular context to initiate cancer presents an interesting contrast with other oncogenic transcription factors such as MYC that act broadly regardless of cell type or tissue. ERG biology in normal tissues provides some precedent. The only tissue that displays absolute ERG-dependence is vascular endothelium, with a phenotype of embryonic lethality in conditional knockout mice due to defects in angiogenesis and blood vessel integrity[35-38]. ERG also plays a context-specific role in hematopoietic stem cells (HSC) by coordinating the balance of self-renewal versus differentiation[39-41]. The context specificity of ERG also extends to human cancer, where recurrent oncogenic translocations are limited to prostate cancer (TMPRSS2-ERG), acute myeloid leukemia (FUS/TLS-ERG) and Ewing's sarcoma (EWS-ERG). Ewing's sarcoma is of particular interest due to challenges in developing ERG-driven GEMMs despite using a range of Cre drivers[42], again indicative of exquisite context dependence. Our discovery of a novel chromatin landscape in prostatic IM cells following ERG induction provides a mechanism by which ERG might acquire oncogenic potential by creating a cell type-specific chromatin context in which it can now act. Whether ERG alone can shape this context (as a pioneer-like TF) or in partnership with another TFs remains to be determined.

Supplementary Note 8. Study Limitations.

Our study has the following limitations. First, while we took orthogonal approaches for lineage tracing, we cannot fully exclude the influences of the differences among Cre drivers (for example the different recombination efficiencies of K8- vs Nkx3-1-$CreER^{T2}$). Future work using

dual recombinase systems will provide more definitive answers. Interestingly, this approach has been applied in a recent study to address cell of origin in the Pten-deletion-only setting which led to the consistent discovery of the Basal^{Lum} population (named Basal-B in the study)[43]. Second, tumors were initiated upon early adulthood (2 months old) in our lineage tracing and transplantation studies. It will be interesting to determine whether aging might affect the cell context dependencies of ERG oncogenicity. Finally, because our study was not designed to thoroughly compare phenotypes across prostate lobes, we cannot fully exclude the possibility of lobe-specific differences in cell of origin preference.

Supplementary Fig. 1

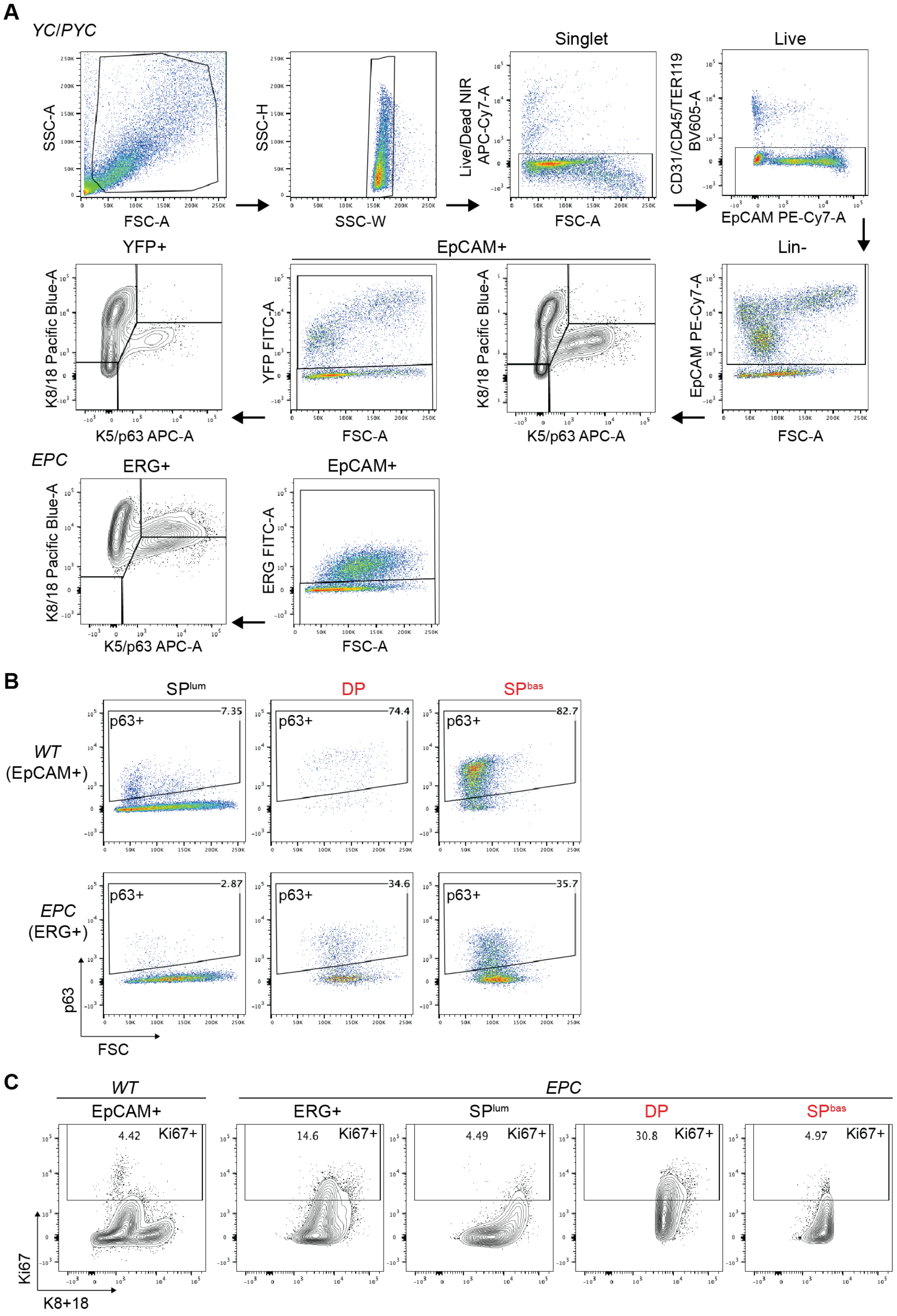

Supplementary Fig. 1. Flow cytometry panel design.

(**A**) Single cell suspension of dissociated prostate cells were gated for debris elimination, singlets (SSC-H vs -W), live cells (Live/Dead NIR exclusion), endothelial/immune exclusion (Lin-, CD31-,CD45-,Ter119-) and epithelial cells (EpCAM+). The epithelial population were further gated on the Cre-recombined population (YFP+ for *YC*/*PYC*, ERG+ for *EPC*) before analyzing basal (K5/p63 single positive, SP^{bas}), luminal (K8/18 single positive, SP^{lum}) and double-positive (DP) populations. DP population is gated as the outlier population based on the *WT* contour plot, assuming that DP cells rarely exist in normal adult prostates. The continuous population of SP^{bas} and DP in tumor samples (*PYC*/*EPC*) are combined and named as IM. (**B**) To assess p63 expression, epithelial or ERG/YFP+ cells were first gated as in **A**, before applying the p63+ gate to populations of interest. (**C**) To assess Ki67 expression, epithelial or ERG/YFP+ cells were first gated as in **A**. Ki67-positive cells were gated as the outlier population based on the *WT* contour plot, assuming that normal adult prostates are rarely proliferative, before applying to other populations of interest.

Supplementary References

1. Lu, T.L. *et al.* Conditionally ablated Pten in prostate basal cells promotes basal-to-luminal differentiation and causes invasive prostate cancer in mice. *Am J Pathol* **182**, 975-91 (2013).
2. Rock, J.R. *et al.* Basal cells as stem cells of the mouse trachea and human airway epithelium. *Proc Natl Acad Sci U S A* **106**, 12771-5 (2009).
3. Van Keymeulen, A. *et al.* Distinct stem cells contribute to mammary gland development and maintenance. *Nature* **479**, 189-93 (2011).
4. Tate, T. *et al.* Pparg signaling controls bladder cancer subtype and immune exclusion. *Nat Commun* **12**, 6160 (2021).
5. Blanpain, C. Tracing the cellular origin of cancer. *Nat Cell Biol* **15**, 126-34 (2013).
6. Bhatia-Gaur, R. *et al.* Roles for Nkx3.1 in prostate development and cancer. *Genes Dev* **13**, 966-77 (1999).
7. Ousset, M. *et al.* Multipotent and unipotent progenitors contribute to prostate postnatal development. *Nat Cell Biol* **14**, 1131-8 (2012).
8. Wang, Z.A. *et al.* Lineage analysis of basal epithelial cells reveals their unexpected plasticity and supports a cell-of-origin model for prostate cancer heterogeneity. *Nat Cell Biol* **15**, 274-83 (2013).
9. Chan, J.M. *et al.* Lineage plasticity in prostate cancer depends on JAK/STAT inflammatory signaling. *Science* **377**, 1180-1191 (2022).
10. Basilio, J. *et al.* Antagonistic Functions of Androgen Receptor and NF-kappaB in Prostate Cancer-Experimental and Computational Analyses. *Cancers (Basel)* **14**(2022).
11. Riedel, M. *et al.* Targeting AP-1 transcription factors by CRISPR in the prostate. *Oncotarget* **12**, 1956-1961 (2021).
12. Koul, D., Yao, Y., Abbruzzese, J.L., Yung, W.K. & Reddy, S.A. Tumor suppressor MMAC/PTEN inhibits cytokine-induced NFkappaB activation without interfering with the IkappaB degradation pathway. *J Biol Chem* **276**, 11402-8 (2001).
13. Gustin, J.A., Maehama, T., Dixon, J.E. & Donner, D.B. The PTEN tumor suppressor protein inhibits tumor necrosis factor-induced nuclear factor kappa B activity. *J Biol Chem* **276**, 27740-4 (2001).
14. Dan, H.C. *et al.* Akt-dependent regulation of NF-kappaB is controlled by mTOR and Raptor in association with IKK. *Genes Dev* **22**, 1490-500 (2008).
15. Mayo, M.W. *et al.* PTEN blocks tumor necrosis factor-induced NF-kappa B-dependent transcription by inhibiting the transactivation potential of the p65 subunit. *J Biol Chem* **277**, 11116-25 (2002).
16. Koul, D. *et al.* PTEN down regulates AP-1 and targets c-fos in human glioma cells via PI3-kinase/Akt pathway. *Mol Cell Biochem* **300**, 77-87 (2007).
17. Muller, M.R. & Rao, A. NFAT, immunity and cancer: a transcription factor comes of age. *Nat Rev Immunol* **10**, 645-56 (2010).
18. Philips, R.L. *et al.* The JAK-STAT pathway at 30: Much learned, much more to do. *Cell* **185**, 3857-3876 (2022).
19. Wang, H.Q. *et al.* STAT3 pathway in cancers: Past, present, and future. *MedComm (2020)* **3**, e124 (2022).

20. Bishop, J.L., Thaper, D. & Zoubeidi, A. The Multifaceted Roles of STAT3 Signaling in the Progression of Prostate Cancer. *Cancers (Basel)* **6**, 829-59 (2014).
21. Morris, D.S., Tomlins, S.A., Montie, J.E. & Chinnaiyan, A.M. The discovery and application of gene fusions in prostate cancer. *BJU Int* **102**, 276-82 (2008).
22. Nam, R.K. *et al.* Expression of TMPRSS2:ERG gene fusion in prostate cancer cells is an important prognostic factor for cancer progression. *Cancer Biol Ther* **6**, 40-5 (2007).
23. Nam, R.K. *et al.* Expression of the TMPRSS2:ERG fusion gene predicts cancer recurrence after surgery for localised prostate cancer. *Br J Cancer* **97**, 1690-5 (2007).
24. Perner, S. *et al.* TMPRSS2-ERG fusion prostate cancer: an early molecular event associated with invasion. *Am J Surg Pathol* **31**, 882-8 (2007).
25. Boormans, J.L. *et al.* Expression of the androgen-regulated fusion gene TMPRSS2-ERG does not predict response to endocrine treatment in hormone-naive, node-positive prostate cancer. *Eur Urol* **57**, 830-5 (2010).
26. Hermans, K.G. *et al.* Overexpression of prostate-specific TMPRSS2(exon 0)-ERG fusion transcripts corresponds with favorable prognosis of prostate cancer. *Clin Cancer Res* **15**, 6398-403 (2009).
27. Habbe, N. *et al.* Spontaneous induction of murine pancreatic intraepithelial neoplasia (mPanIN) by acinar cell targeting of oncogenic Kras in adult mice. *Proc Natl Acad Sci U S A* **105**, 18913-8 (2008).
28. Gidekel Friedlander, S.Y. *et al.* Context-dependent transformation of adult pancreatic cells by oncogenic K-Ras. *Cancer Cell* **16**, 379-89 (2009).
29. Molyneux, G. *et al.* BRCA1 basal-like breast cancers originate from luminal epithelial progenitors and not from basal stem cells. *Cell Stem Cell* **7**, 403-17 (2010).
30. Lim, E. *et al.* Aberrant luminal progenitors as the candidate target population for basal tumor development in BRCA1 mutation carriers. *Nat Med* **15**, 907-13 (2009).
31. Van Keymeulen, A. *et al.* Reactivation of multipotency by oncogenic PIK3CA induces breast tumour heterogeneity. *Nature* **525**, 119-23 (2015).
32. Koren, S. *et al.* PIK3CA(H1047R) induces multipotency and multi-lineage mammary tumours. *Nature* **525**, 114-8 (2015).
33. Juul, N.H. *et al.* KRAS(G12D) drives lepidic adenocarcinoma through stem-cell reprogramming. *Nature* **619**, 860-867 (2023).
34. Ferone, G., Lee, M.C., Sage, J. & Berns, A. Cells of origin of lung cancers: lessons from mouse studies. *Genes Dev* **34**, 1017-1032 (2020).
35. Vijayaraj, P. *et al.* Erg is a crucial regulator of endocardial-mesenchymal transformation during cardiac valve morphogenesis. *Development* **139**, 3973-85 (2012).
36. Birdsey, G.M. *et al.* The endothelial transcription factor ERG promotes vascular stability and growth through Wnt/beta-catenin signaling. *Dev Cell* **32**, 82-96 (2015).
37. Ginsberg, M. *et al.* Efficient direct reprogramming of mature amniotic cells into endothelial cells by ETS factors and TGFbeta suppression. *Cell* **151**, 559-75 (2012).
38. Han, R., Pacifici, M., Iwamoto, M. & Trojanowska, M. Endothelial Erg expression is required for embryogenesis and vascular integrity. *Organogenesis* **11**, 75-86 (2015).
39. Knudsen, K.J. *et al.* ERG promotes the maintenance of hematopoietic stem cells by restricting their differentiation. *Genes Dev* **29**, 1915-29 (2015).
40. Loughran, S.J. *et al.* The transcription factor Erg is essential for definitive hematopoiesis and the function of adult hematopoietic stem cells. *Nat Immunol* **9**, 810-9 (2008).

41. Wilson, N.K. *et al.* Combinatorial transcriptional control in blood stem/progenitor cells: genome-wide analysis of ten major transcriptional regulators. *Cell Stem Cell* **7**, 532-44 (2010).
42. Minas, T.Z. *et al.* Combined experience of six independent laboratories attempting to create an Ewing sarcoma mouse model. *Oncotarget* **8**, 34141-34163 (2017).
43. Guo, W. *et al.* JAK/STAT signaling maintains an intermediate cell population during prostate basal cell fate determination. *Nat Genet* **56**, 2776-2789 (2024).
