# Supplementary material for: ERG-driven prostate cancer initiation is cell-context dependent and requires KMT2A and DOT1L
Source: Nat Genet. 2025 Aug 26;57(9):2177–91. doi: 10.1038/s41588-025-02289-w (PMC12425824; doi:10.1038/s41588-025-02289-w)
Supplement: Supplementary file 2 — Reporting Summary [file 41588_2025_2289_MOESM2_ESM.pdf]

nature portfolio

Corresponding author(s): Weiran Feng; Charles Sawyers

Last updated by author(s): Apr 21, 2025

Reporting Summary

Nature Portfolio wishes to improve the reproducibility of the work that we publish. This form provides structure for consistency and transparency in reporting. For further information on Nature Portfolio policies, see our Editorial Policies and the Editorial Policy Checklist.

Statistics

For all statistical analyses, confirm that the following items are present in the figure legend, table legend, main text, or Methods section.

n/a	Confirmed	
☐	☒	The exact sample size (*n*) for each experimental group/condition, given as a discrete number and unit of measurement
☐	☒	A statement on whether measurements were taken from distinct samples or whether the same sample was measured repeatedly
☐	☒	The statistical test(s) used AND whether they are one- or two-sided<br>*Only common tests should be described solely by name; describe more complex techniques in the Methods section.*
☒	☐	A description of all covariates tested
☐	☒	A description of any assumptions or corrections, such as tests of normality and adjustment for multiple comparisons
☐	☒	A full description of the statistical parameters including central tendency (e.g. means) or other basic estimates (e.g. regression coefficient) AND variation (e.g. standard deviation) or associated estimates of uncertainty (e.g. confidence intervals)
☐	☒	For null hypothesis testing, the test statistic (e.g. *F*, *t*, *r*) with confidence intervals, effect sizes, degrees of freedom and *P* value noted<br>*Give P values as exact values whenever suitable.*
☒	☐	For Bayesian analysis, information on the choice of priors and Markov chain Monte Carlo settings
☒	☐	For hierarchical and complex designs, identification of the appropriate level for tests and full reporting of outcomes
☒	☐	Estimates of effect sizes (e.g. Cohen's *d*, Pearson's *r*), indicating how they were calculated

Our web collection on statistics for biologists contains articles on many of the points above.

Software and code

Policy information about availability of computer code

Data collection	no software was used
Data analysis	ArchR : Version 1.0.2<br>ComplexHeatmap: Version 2.20.0<br>Data.table: Version 1.17.0<br>fgsea: Version 1.30.0<br>ggplot2: Version 3.5.1<br>Macs3: Version 3.0.0b1<br>Motifmatchr: Version 1.26.0<br>Presto: Version 1.0.0<br>R: Version 4.4.0<br>Seurat: Version 5.2.1<br>Scanpy: Version 1.10.1<br>FlowJo: 10.9.0

For manuscripts utilizing custom algorithms or software that are central to the research but not yet described in published literature, software must be made available to editors and reviewers. We strongly encourage code deposition in a community repository (e.g. GitHub). See the Nature Portfolio guidelines for submitting code & software for further information.

Data

Policy information about availability of data

All manuscripts must include a data availability statement. This statement should provide the following information, where applicable:

- Accession codes, unique identifiers, or web links for publicly available datasets
- A description of any restrictions on data availability
- For clinical datasets or third party data, please ensure that the statement adheres to our policy

Raw sequencing data are publicly available from the Gene Expression Omnibus: GSE257543 (scRNA-seq), GSE258962 (scATAC-seq), GSE294013 (bulk RNA-seq). Existing scRNA-seq data reanalyzed in our manuscript are available as: GSE176031, GSE181294. All other data are available in the main text or the supplementary materials. Source data are provided with this paper.

Research involving human participants, their data, or biological material

Policy information about studies with human participants or human data. See also policy information about sex, gender (identity/presentation), and sexual orientation and race, ethnicity and racism.

Reporting on sex and gender	n/a
Reporting on race, ethnicity, or other socially relevant groupings	n/a
Population characteristics	n/a
Recruitment	n/a
Ethics oversight	n/a

Note that full information on the approval of the study protocol must also be provided in the manuscript.

Field-specific reporting

Please select the one below that is the best fit for your research. If you are not sure, read the appropriate sections before making your selection.

☒ Life sciences ☐ Behavioural & social sciences ☐ Ecological, evolutionary & environmental sciences

For a reference copy of the document with all sections, see nature.com/documents/nr-reporting-summary-flat.pdf

Life sciences study design

All studies must disclose on these points even when the disclosure is negative.

Sample size	No statistical method was used to predetermine sample size.
Data exclusions	No data were excluded
Replication	Experiments were repeated with a minimum of two independent experiments and noted in figure legends.
Randomization	Age- and litter- matched male mice were either freshly purchased from repository or randomized into different experimental groups used for all animal studies.
Blinding	Histological grading for tumor samples were performed by a blinded prostate cancer pathologist. Other analysis (single cell sequencing, flow cytometry, imaging analysis) were automatedly performed.

Reporting for specific materials, systems and methods

We require information from authors about some types of materials, experimental systems and methods used in many studies. Here, indicate whether each material, system or method listed is relevant to your study. If you are not sure if a list item applies to your research, read the appropriate section before selecting a response.

Materials & experimental systems

n/a	Involved in the study
☐	☒ Antibodies
☐	☒ Eukaryotic cell lines
☒	☐ Palaeontology and archaeology
☐	☒ Animals and other organisms
☐	☒ Clinical data
☒	☐ Dual use research of concern
☒	☐ Plants

Methods

n/a	Involved in the study
☒	☐ ChIP-seq
☐	☒ Flow cytometry
☒	☐ MRI-based neuroimaging

Antibodies

Antibodies used

Antibodies for tissue IHC and IF:
GFP Chicken 2ug/ml ER2 Abcam ab13970 IHC
K5 Rabbit 1ug/ml ER2 Abcam ab53121 IHC
K8/18 Rabbit 0.12ug/ml ER2 Abcam ab53280 IHC
P63 mouse prediluted ER2 Ventana 790-4509 IHC
ERG rabbit 1ug/ml ER2 Epitomics 2805-1 IHC
pAkt Rabbit 1ug/ml ER2 Cell signaling technology 4060 IHC
Trop2 Rabbit 0.5ug/ml ER2 Abcam ab214488 IHC
Nkx3-1 Rabbit 0.44ug/ml ER2 Proteintech 13069-1-AP IHC
GFP Chicken 2ug/ml ER2 Abcam ab13970 IF
K5 Rabbit 0.25ug/ml ER2 abcam ab53121 IF
K8/18 Rabbit 0.03ug/ml ER2 Abcam ab53280 IF
Ar Rabbit 0.5ug/ml ER2 Abcam ab108341 IF
ERG Rabbit 0.5ug/ml ER2 Epitomics 2805-1 IF
pAkt Rabbit 0.5ug/ml ER2 Cell signaling technology 4060 IF

Antibodies for flow cytometry:
K8-AF405 Rabbit 1:500 Abcam ab210139 Primary
K8-AF647 Rabbit 1:500 Abcam ab192468 Primary
K18-Biotin Rabbit 1:500 Abcam ab27553 Primary
K5-APC Rabbit 1:500 Abcam ab224984 Primary
K5-PE Rabbit 1:500 Abcam ab224985 Primary
p63-AF647 Rabbit 1:500 Abcam ab246728 Primary
ERG Rabbit 1:1000 Abcam ab92513 Primary
ERG-AF488 Rabbit 1:500 Abcam ab196374 Primary
ERG-AF647 Rabbit 1:500 Abcam ab196149 Primary
Pten Rabbit 1:500 Abcam ab170941 Primary
Ki67-PE Rat 1:50 BioLegend 652404 Primary
CD45-BV605 Rat 1:600 BioLegend 103139 Primary
CD31-BV605 Rat 1:600 BioLegend 102427 Primary
TER-119-BV605 Rat 1:600 BioLegend 116239 Primary
EpCAM-PE/Cy7 Rat 1:1000 BioLegend 118216 Primary
CD49f-PE Rat 1:200 BD 555736 Primary
CD24-AF647 Rat 1:200 BioLegend 101818 Primary
Rabbit IgG Goat 1:1000 Thermo Fisher A-21245 Secondary
Biotin Streptavidin-AF405 1:1000 Thermo Fisher S32351 Secondary

Antibodies for Western Blot:
Kmt2a (1:1,000; Cell Signaling Technology 14689), Menin (1:1,000; Cell Signaling Technology 6891S), Hsp90 (1:1,000; Cell Signaling Technology 4877S), Actin-HRP (horseradish peroxidase) (1:10,000; Abcam ab49900)

Validation

Antibodies were all validated according to manufacturer's website:
Abcam (https://www.abcam.com); Cell Signaling Technology (https://www.cellsignal.com); Thermo Fisher Scientific (https://www.thermofisher.com); BioLegend (https://www.biolegend.com/); Ventana (https://diagnostics.roche.com/global/en/products/product-category/product-finder.html?limit=18&tags=%5B%7B%22tags_es%22%3A%22Product%20Families%3AVENTANA%22%7D%5D&facets=%22familyl1%3AVENTANA%22&listing=Products&);
Validation was performed for human (ERG) and/or mouse (other protein targets) species using applications corresponding to those used in this study: Flow cytometry, IHC/IF, Western Blot.

Eukaryotic cell lines

Policy information about cell lines and Sex and Gender in Research

Cell line source(s)

Mouse prostate organoids were derived from mice with the appropriate genotypes.

Authentication	No authentication was performed since the organoid lines were freshly derived from mice with the appropriate genotypes that have been confirmed.
Mycoplasma contamination	All cells were tested negative for mycoplasma contamination.
Commonly misidentified lines (See ICLAC register)	N/A

Animals and other research organisms

Policy information about studies involving animals; ARRIVE guidelines recommended for reporting animal research, and Sex and Gender in Research

Laboratory animals	Mice were in NSG, or mixed strain backgrounds. Males and females were used for breeding. Males were used for study since the focus is on prostate. Mice were maintained under 12h light/dark cycle (switching at 6am/pm), with controlled temperature and humidity, and with access to regular chow and sterilized water.
Wild animals	The study did not involve wild animals
Reporting on sex	The study only applies to males since the focus is on prostate.
Field-collected samples	The study did not involve field-collected samples.
Ethics oversight	Mouse experiments were conducted under protocol 06-07-012 approved by the Institutional Animal Care and Use Committee of Memorial Sloan Kettering Cancer Center (MSKCC), New York.

Note that full information on the approval of the study protocol must also be provided in the manuscript.

Clinical data

Policy information about clinical studies
All manuscripts should comply with the ICMJE guidelines for publication of clinical research and a completed CONSORT checklist must be included with all submissions.

Clinical trial registration	N/A
Study protocol	N/A
Data collection	All clinical data from the study were obtained from publicly available dataset (listed in Method section).
Outcomes	N/A

Plants

Seed stocks	N/A
Novel plant genotypes	N/A
Authentication	N/A

Flow Cytometry

Plots

Confirm that:

☒ The axis labels state the marker and fluorochrome used (e.g. CD4-FITC).

☒ The axis scales are clearly visible. Include numbers along axes only for bottom left plot of group (a 'group' is an analysis of identical markers).

☒ All plots are contour plots with outliers or pseudocolor plots.

☒ A numerical value for number of cells or percentage (with statistics) is provided.

Methodology

Sample preparation	Prostate organoid cells or freshly dissociated prostate tissue cells were stained with surface marker antibodies prior to sorting, for fixed and permeabilized for intracellular staining prior to analysis, as detailed in Methods section.
Instrument	BD Fortessa or MACSQuant 16 for analysis, BD Aria or Symphony for sorting.
Software	FlowJo
Cell population abundance	Sorted population was > 85% pure as determined by post-sort analysis
Gating strategy	Cells were gated on FSC/SSC for debris elimination, singlets (SSC-H vs -W), live cells (Live/Dead NIR exclusion) prior to downstream analysis. Gating were based on either biological (ie non-expressing cells) or technical (ie FMO) negative controls.

☒ Tick this box to confirm that a figure exemplifying the gating strategy is provided in the Supplementary Information.
