# Supplementary figures and images for: ERG-driven prostate cancer initiation is cell-context dependent and requires KMT2A and DOT1L
Source: Nat Genet. 2025 Aug 26;57(9):2177–91. doi: 10.1038/s41588-025-02289-w (PMC12425824; doi:10.1038/s41588-025-02289-w)

Extended Data Fig. 10a

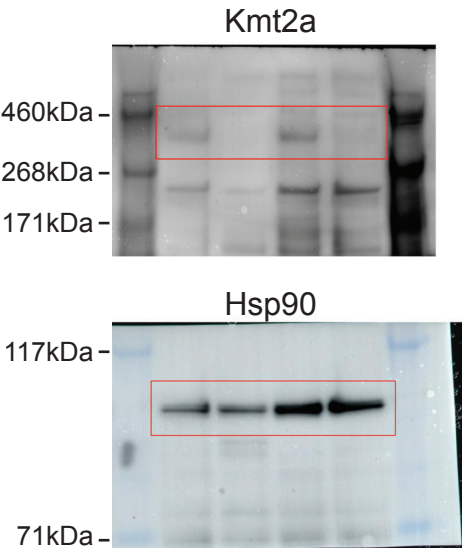

Extended Data Fig. 10d

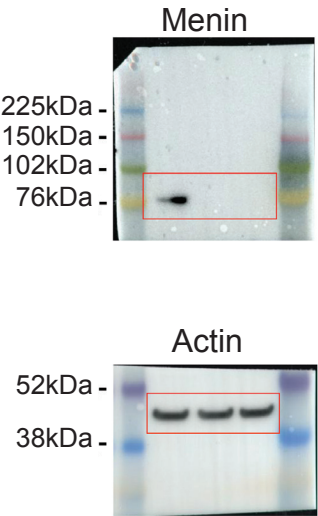

Supplement: Supplementary file 16 — Unprocessed western blots. [file 41588_2025_2289_MOESM16_ESM.pdf]
